# PI3K(p110α) as a determinant and gene therapy for atrial enlargement in atrial fibrillation

**DOI:** 10.1007/s11010-022-04526-w

**Published:** 2022-07-28

**Authors:** Martin Ezeani, Sandeep Prabhu

**Affiliations:** 1 NanoBiotechnology Laboratory, Central Clinical School, Australian Centre for Blood Diseases, Monash University, Melbourne, VIC 3004 Australia; 2 The Alfred, and Baker Heart and Diabetes Institute, Melbourne, Australia; 3 The University of Melbourne, Melbourne, Australia

**Keywords:** PI3K(p110α), Mouse model, Prevention, Gene therapy, Atrial enlargement, Atrial fibrillation

## Abstract

Atrial fibrillation (AF) is an irregular heart rhythm, characterised by chaotic atrial activation, which is promoted by remodelling. Once initiated, AF can also propagate the progression of itself in the so-called ‘‘AF begets AF’’. Several lines of investigation have shown that signalling molecules, including reactive oxygen species, angiotensin II, and phosphoinositide 3-kinases (PI3Ks), in presence or absence of cardiovascular disease risk factors, stabilise and promote AF maintenance. In particular, reduced cardiac-specific PI3K activity that is not associated with oncology is cardiotoxic and increases susceptibility to AF. Atrial-specific PI3K(p110α) transgene can cause pathological atrial enlargement. Highlighting the crucial importance of the p110α protein in a clinical problem that currently challenges the professional health care practice, in over forty (40) transgenic mouse models of AF (Table1), currently existing, of which some of the models are models of human genetic disorders, including PI3K(p110α) transgenic mouse model, over 70% of them reporting atrial size showed enlarged, greater atrial size. Individuals with minimal to severely dilated atria develop AF more likely. Left atrial diameter and volume stratification are an assessment for follow-up surveillance to detect AF. Gene therapy to reduce atrial size will be associated with a reduction in AF burden. In this overview, PI3K(p110α), a master regulator of organ size, was investigated in atrial enlargement and in physiological determinants that promote AF.

**Table 1:** Transgenic and Knockout Mouse Models of AF

	**Gene Alteration**	**Atrial enlargement**	**Fibrosis**	**Thrombus**	**Ventricular dysfunction based on echo and/or catheter**	**Conduction abnormalities by ECG**	**APD Alteration**	**AF pattern/other major cellular and molecular mechanisms**	**References**
Rho GDIα TG	Cardiac-specific overexpression of Rho GDP dissociation inhibitor (GDI)α with α-myosin heavy chain (α-MHC) promoter	Atrial weight 0.6-fold increase vs NTg at 4 months but no changes at 4 weeks	✔ no significant increase in atrial and ventricle	Not reported	↔	Sinus bradycardia, varying degrees of AV block, prolongation of P-wave duration, and PR interval at 7 months	Not reported	Spontaneous Other mechanisms oreduced Connexin 40 expression oincreased expression of RhoA, Rac1, and Cdc42	[58]
RhoA	Cardiac-specific overexpression of RhoA with α-MHC promoter	Atrial weight threefold increase vs NTg	✔ in ventricle	Not reported	✔	Bradycardia and AV block	Not reported	Spontaneous Other mechanisms oincreased expression of hypertrophic genes oInflammation	[59]
Junction TG	Cardiac-specific overexpression of junctin protein with α-MHC promoter	Atrial weight, more than tenfold increase vs WT for right atrium	✔ in atrial and ventricle	✔ in left and right atria	✔	Bradycardia	Atrial and ventricle APD_70,_ phase 3 ↑	Spontaneous Other mechanisms oreduced triadin, RYR2, diastolic Ca^2+^, and Ca^2+^ transient amplitude	[60]
Junctate 1 TG	Cardiac-specific SR-located Ca^2+^-binding protein junctate 1 overexpression with α-MHC promoter	Atrial weight, fourfold increase for left atrium and about fivefold increase for right atrium vs WT	↑ in atria and ventricle	✔ Intra-atrial thrombi	✔	Ventricular bigeminy, sinus pause, and bradycardia	APD_90,_ phase 4 ↑	Spontaneous Other mechanisms oreduced phospholamban phosphorylation, troponin I phosphorylation, Calreticulin, and RyR2 channel oreduced SR Ca^2+^ content, Ca^2+^ transient amplitude oincreased I_Ca,L_	[61]
AMPK TG^N488I^	Cardiac-specific PRKAG2 (AMPK γ2 subunit) overexpression with missense mutation	Not reported	Not reported	Not reported	✔	Reduced PR interval, persistent sinus bradycardia without AV block	Not reported	Spontaneous and paroxysmal Other mechanisms ocardiac glycogen accumulation	[62]
A_1_AR TG	Cardiac-specific overexpression of A_1_ adenosine receptor (A_1_AR) with α-MHC	No difference	No fibrosis	Not reported	✔	Slow AV conduction	APD_90,_ phase 4 ↔ APD_50,_ phase 2 ↔ APD_70,_ phase 2 ↔	Spontaneous	[63]
A_3_tg TG	Cardiac-specific overexpression of A_3_ adenosine receptor (A_3_AR) with α-MHC promoter	Atrial size onefold and twofold increase at 12 weeks and 21 weeks, respectively, vs NTg	Not present in atria and ventricle	Not reported	✔	Absence of normal sinus rhythm, bradycardia, and intermittently complete	Not reported	Spontaneous Other mechanisms oreduced SERCA mRNA levels	[64]
RTEF1 TG	Cardiac-specific overexpression of Transcription enhancer factor-1-related factor (RTEF1) with α-MHC promoter	Atrial weight 4–sixfold increase vs control	Not present in atria and ventricle	✔ Organised	Not reported	Slow conduction in working myocardium, prolonged PR interval, and QRS duration	Not reported	Spontaneous Mechanisms oincreased PP1β phosphatase ochronic dephosphorylation of cardiac connexin	[65]
ACE 8/8 TG	Cardiac-restricted angiotensin-converting enzyme (ACE) Overexpression with α-MHC Ang II concentration was 4.3-fold higher in ACE mice compared to WT	Atrial weight, about threefold increase vs WT	✔ in atria but not in ventricle	Not reported	✔	AV block	Not reported	Spontaneous	[66]
K_ir_2.1 TG	K_ir_2.1 *I*_K1_ channel subunit cardiac-specific overexpression with α-MHC promoter	Atrial weight, left and right atrial to body weight 65% and 141% increase, respectively, vs control	Not reported	Not reported	✔	Absence of T wave and reduced QT interval	APD_90,_ phase 4 ↓ APD_50,_ phase 2 ↔ APD_75,_ phase 3 ↔ MAP90 Phase 4 ↓ MAP75 phase 3 ↓ MAP50_,_ phase 2 ↔	Spontaneous	[67]
*Kcne1*^−/−^	K^+^-channel KCNE1 subunit global protein deletion in mouse	Normal atrial size	Not present in atria and ventricle	Not reported	↔	AV block	**A**PD_50,_ phase 2 ↓ APD_90,_ phase 4 ↓	Spontaneous	[68]
hKCNE1-hKCNQ1 TG	Human (h)KCNE1-hKCNQ1 Cardiac-specific overexpression with α-MHC promoter in mouse	Not reported	Not reported	Not reported	Not reported	Complex atrial and irregular ventricular excitation	β-AR mediated APD_50,_ phase 2 ↑ APD_90,_ phase 4 ↓	Spontaneous Other mechanisms oIncreased I_Ks_ density	[69]
*Des*^*−/−*^	Desmin global knockout	Not reported	Not reported	Not reported	Not reported	Supraventricular premature beats, spontaneous ventricular premature beats, and Wenckebach periodicity	Not reported	Spontaneous Other mechanisms oHypokalemia, oReduced refractory period	[70]
CREM-IbΔC-X	Human cAMP-response element modulator (CREM) heart-directed overexpression with α-MHC promoter	Atrial weight, about 5–sevenfold increase vs NTg at 12–16 weeks	Not present in left atrium and ventricle	✔ Organised thrombi in left and right atria	✔	Not reported	Not reported	Spontaneous Other mechanisms oReduced phosphorylation of CREB and of PLB oIncreased phosphorylation of SERCA2, PP1, and mRNA levels of ANP	[71]
CREM-IbΔC-X	Human cAMP-CREM heart-directed Overexpression with α-MHC promoter	Left atrial size, twofold increase vs WT at 13–17 weeks	↑ in atria	Not reported	Not reported	Ectopic beats	APD_25,_ phase 1 ↑ APD_50,_ phase 2 ↑ APD_90_ phase 4 ↑	Spontaneous and persistent Other mechanisms oLeaky SR Ca^2+^ stores oDownregulation of connexin 40	[72]
CREM-IbΔC-X	Human cAMP- CREM and reduced RyR_2_-S2814A phosphorylation heart-directed overexpression with germline transmission and Meox2-Cre crossing	Atrial weight, sixfold increase vs WT at 3 months	↑ in atria and ventricle	Not reported	↔	Spontaneous atrial ectopy	APD_80,_ phase 4 ↑	Spontaneous at 3-month paroxysmal and persistent at 4–5 months Other mechanisms oincreased SR Ca^2+^ leak and CaMKII activity oreduced connexin 40	[73]
JDP TG	Heart-restricted c-Jun dimerization protein 2 overexpression with α-MHC promoter	Atrial cell diameter 1.4-fold increase vs WT	Not present in the atrial and ventricle	Not reported	↔	Increased PR interval, AV block and Wenckebach periodicity	Not reported	Spontaneous Other mechanisms oreduced expression of connexin 40 and 43 oAng II signalling	[74]
RacET	Heart-restricted constitutively active Rac1 Rho GTPase overexpression with α-MHC promoter	Atrial weight, fourfold increase vs WT	↑ in atria and ventricle	Not reported	✔	No observable conduction defects except AF	Not reported	Spontaneous and persistent Other mechanisms oincreased NADPH oxidase activity	[75]
Anxa7^−/−^	Annexin global knockout	Not reported	Not reported	Not reported	↔ at basal	AV block, ventricular tachyarrhythmia, shorter P-wave and QRS duration, and abnormal conduction velocity	Not reported	Spontaneous Other mechanisms oreduced protein expression of SERCA2a oincrease expression of NCX protein oβ_1_-adrenergic signalling	[76]
TNF1.6 TG	Heart-directed overexpression of tumour necrosis factor-α with α-MHC promoter	Isolated atrial area 3.6-fold increase from 6 to 9 months in female vs NTg	✔ in atria	✔ Organised thrombi in atria	Not reported	Episodes of second degree AV block, premature beats, and Ventricular ectopy	APD_75_ Phase 4 ↔	Spontaneous Other mechanisms oimpaired Ca^2+^ loading oreduced intracellular Ca^2+^ transients	[77]
MHCsTNF TG	Cardiac-specific overexpression of tumour necrotic factor with α-MHC promoter	Not reported	Not reported	Not reported	✔	AV junctional rhythm, short PR interval and wide QRS complex	Not reported	Spontaneous Other mechanisms oreduced connexion 40 expression oinflammation	[78]
MURCTG	Cardiac-specific overexpression of muscle-related coiled-coil protein with α-MHC promoter	Enlarged atrial compared to NTg	↑ in atria and ventricle	Thrombus in the left atrial	✔	Complete AV block and prolongation of the PR interval	Not reported	Spontaneous Other mechanisms oreduced SERCA2, increased ANP, BNP, βMHC, TGF-β1, TGF-β2, and TGF-β3	[79]
*Nup155*^±^	Reduced nuclear envelope permeability by nucleoporin (NUP) 155 gene missense mutation on R391H	Not reported	Not reported	Not reported	Not reported	Irregular RR intervals	APD_90,_ phase 4 ↓	Spontaneous Other mechanisms oreduced HSP70 nuclear localization	[80]
*a1D*^*−/−*^	L-type Ca^2+^ channel (Ca_v_1.3) subunit global knockout	Not reported	Not reported	Not reported	Not reported	SA and AV nodes conduction defects	Not reported	Spontaneous Other mechanisms olack of Ca_v_1.3, and reduced I_Ca,L_	[81]
LTCC (α1D^−/−^)	L-type Ca^2+^ channel α1D subunit global knockout	Smaller compared with WT	Not reported	Not reported	Not reported	Sinus bradycardia and AV block	Not reported	Spontaneous Other mechanisms oreduced I_Ca,L_, Ca^2+^ transient amplitude, and SR Ca^2+^ content	[82]
dnPI3K-DCM	Cardiac-specific dominant negative phosphoinositide 3-kinase p110α (dnPI3K) DCM due to overexpression of mammalian sterile 20-like kinase 1 expression with α-MHC promoter	Atrial size 3.45-fold increase vs NTg	↑ in atria and ventricle	✔ Chronic thrombi in the left atrium	✔	Prolonged PR intervals, double peak P-wave, and second and third degree AV block	Not reported	Spontaneous Other mechanisms oaltered expression of metabolic genes and K^+^ channels oreduced HSP70	[16]
*Dct*^−/−^	Melanin synthesis enzyme dopachrome tautomerase global knockout	Not reported	No	Not reported	↔	No observable conduction defects except for AF	APD_50_, phase 2 ↔ APD_90_, phase 4 ↔	Spontaneous Other mechanisms oplasma membrane caveolae accumulation oenlargement of mitochondria	[83]
*RyR*2^R176Q/+^	R176Q mutation in RYR2 gene through germline transmission and Meox2-Cre crossing	Normal atrial size	No fibrosis in atrial and ventricle	Not reported	Not reported	RR interval variability, absence of P-wave	APD_50_ phase 2 ↔ APD_80_ phase 4 ↔	Spontaneous Other mechanisms oincreased CaMKII-dependent phosphorylation of RyR2 oelevated SR Ca^2+^ leak	[84]
Gα_q_ TG	Overexpression of activated Gαq cardiac protein with α-MHC promoter	Left atrial size, 2.5-fold increase vs WT	↑ in atria but not in ventricle	✔ Left atrial, unorganised thrombus	Not reported	Premature atrial contraction and irregular RR interval	APD_80_, phase 4 ↑	Spontaneous	[85]
NppaCre^+^Pitx2^−^/^−^	Atrial and ventricular-restricted loss of function of paired-like homeodomain transcription factor 2 (PITX2)	Atrial length about 1.6-fold increase for left atrium and 1.2-fold increase for right atrium vs WT	↑ in ventricle but not in atria	Not reported	Not reported	AV block	APD_20_ phase 1, ↔ APD_50_ phase 2, ↔ APD_90_ phase 4, ↔	Spontaneous Other mechanisms oreduced expression of Pitx2, oreduced expression of Nav1.5 oreduced expression of Kir2.1	[86]
AnkB^±^	Ankyrin-B (ANK2) heterologous null mutation	Not reported	Not reported	Not reported	✔	Spontaneous bradycardia and abnormal ventricular response	APD_90_ phase 4, ↓	Spontaneous Other mechanisms oreduced I_Ca,L_ oreduced Cav1.3 expression, osignalling interaction between ankyrin-B and Cav1.2	[87]
D1275N-Na_v_1.5	Human sodium channel Na_v_1.5 global missense mutation	Not reported	No	Not reported	✔	prolongation of P-wave and QRS duration PR interval and AV block	APD_50_, phase 2 ↑ APD_90_, phase 4 ↑	Spontaneous Other mechanisms oreduced peak I_Na_ oincreased late I_Na_	[88]
SLN^−/−^	Sarcolipin global knockout	No difference	↑ in atria but not in ventricle	Not reported	Not reported	Small oscillatory waves	APD_50_, phase 2 ↔ APD_90_, phase 4 ↑	Spontaneous Other mechanisms oSR Ca^2+^ overload oDADs oincreased phosphorylation of RyR_2_	[89]
FKBP12.6^−/−^	FK506-binding protein deficiency with reduced RYR2 phosphorylation at S2814	Not reported	Not reported	Not reported	Not reported	Absence of P-waves and irregular RR intervals	APD_30_, phase 2 ↔ APD_50_, phase 2 ↔	Spontaneous Other mechanisms oLack of FK506-binding protein 12.6 oDADs oSR Ca^2+^ leak oincreased I_NCX_ oCaMKII phosphorylation of RYR_2_ and PLB	[90]
MHC-TGFcys^33^ser	Cardiac-restricted constitutively active TGFβ1 overexpression with αMHC promoter	Not reported	↑ in atria	Not reported	Not reported	Activation wavefront	APD_80_, phase 4 ↓ for both left and right atria	Spontaneous Other mechanisms oincreased Ca^2+^ transient	[91]
DN-MSTN TG13 TG	Heart-directed overexpression of the N-terminal pro-peptide with α-MHC promoter	Atrial weight 3.7-fold increase vs NTg	↑ in atria	Appears present	↔	AV block, Bradycardia Increased P-waves and QRS duration	Not reported	Spontaneous Other mechanisms oreduced connexin 40 expression	[92]
*Casq2*^−/−^	Calsequestrin 2 global knockout	Atria tissue area, about 1.8–2.0-fold increase vs WT	No differences	Not reported	✔	Atrial ectopic activity, bradycardia	APD_80,_ phase 4↑	Spontaneous	[93]
LKB1 knockout	Cardiac-specific AMPK-activating liver kinase B1 (LKB1) knockout with α-MHC promoter	Atria size, about twofold increase for paroxysmal at 4–6 weeks and threefold increase for persistent AF over 6 weeks vs WT	↑ in atria	✔ Intra-atrial thrombi	↔	Increased PR interval and QRS duration in paroxysmal AF	Not reported	Paroxysmal and persistent Other mechanisms oreduced expression of AMPK oincreased in connexin 40 and 43 expression oROS and inflammation	[94]
F1759A-Na_v_1.5-dTG	Human sodium channel Na_v_1.5 cardiac-specific expression with α-MHC promoter	Right and left atria area increase by 52% and 54%, respectively, vs control	↑ in atria and ventricle	Not reported	✔	Premature ventricular complexes and non-sustained polymorphic VT	APD_80,_ phase 4 ↑ for both right and left atria	Spontaneous Other mechanisms oincreased late I_Na_ oincreased glycogen accumulation omyofibril disorganisation omitochondria injury oNCX regulation of Na^+^ entry	[95]
LKB1/CTR	LKB1/CT atrial-specific knockdown	Not reported	↑ in atria	Not reported	↔	Irregularly irregular R–R intervals	Not reported	Spontaneous Other mechanisms oAtrial cardiomyocyte produces calcitonin oCalcitonin receptor and its ligand signalling governs fibroblast roles oParacrine signalling between atrial cardiomyocyte released calcitonin and fibroblast	[96]
PLK2 deficiency	PLK2 Knockout	Greater left atrial area	↑ in atria	Not reported	↔	ventricular tachycardia	APD ↔ ERP ↔	Spontaneous Other mechanisms oPLK2/ERK/OPN is a dominant structural remodelling axis for AF generation	[97]

Mouse models that have been used to study the pathophysiology of AF, including atrial enlargement, electrophysiological alterations, apoptosis, functional and molecular underpinnings, and anatomical, transgenic; RYR2, ryanodine receptor 2; SR, sarcoplasmic reticulum; APD, action potential; SERCA mRNA, sarco/endoplasmic reticulum Ca^2+^-ATPase messenger ribonucleic acid; CTR, calcitonin receptor; KCNE1, potassium voltage-gated channel subfamily E member 1; AV, Atrioventricular block; MAP, monophasic action potential; PLB, phospholamban; ANP, atrial natriuretic peptide; β-AR, beta adrenergic receptor; PPβ1, protein phosphatase type 1β; NADPH, nicotinamide adenine dinucleotide phosphate; CaMKII, Ca^2+^/calmodulin-dependent protein kinase II; NCX, sodium–calcium exchanger; SERCA2a, Sarco/endoplasmic reticulum calcium (Ca^2+^) ATPase gene; TGF- β, Transforming growth factor beta; BNP, brain natriuretic peptide; HSP70, heat shock protein 70; DCM, dilated cardiomyopathy; AMPK, 5' adenosine monophosphate-activated protein kinase; PLK2, polo-like kinase 2; OPN, osteopontin; ERK1/2, extracellular signal-regulated kinase ½. ↔ unchanged in that condition; ✔ present in that condition; ↑ increased in that condition; ↓ reduced in that condition

Transgenic and Knockout Mouse Models of AF

Spontaneous

Other mechanisms

oreduced Connexin 40 expression

oincreased expression of RhoA, Rac1, and Cdc42

✔ in

ventricle

Spontaneous

Other mechanisms

oincreased expression of hypertrophic genes

oInflammation

Atrial and ventricle APD_70,_

phase 3 ↑

Spontaneous

Other mechanisms

oreduced triadin, RYR2, diastolic Ca^2+^, and Ca^2+^ transient amplitude

Cardiac-specific SR-located Ca^2+^-binding protein

junctate 1 overexpression with α-MHC promoter

Spontaneous

Other mechanisms

oreduced phospholamban phosphorylation, troponin I phosphorylation, Calreticulin, and RyR2 channel

oreduced SR Ca^2+^ content, Ca^2+^ transient amplitude

oincreased I_Ca,L_

Reduced PR interval,

persistent sinus bradycardia without AV block

Spontaneous and paroxysmal

Other mechanisms

ocardiac glycogen accumulation

APD_90,_ phase 4 ↔

APD_50,_

phase 2 ↔

APD_70,_

phase 2 ↔

Absence of normal sinus rhythm, bradycardia, and intermittently

complete

Spontaneous

Other mechanisms

oreduced SERCA mRNA levels

Cardiac-specific overexpression of Transcription enhancer factor-1-related factor

(RTEF1) with α-MHC promoter

Atrial weight

4–sixfold increase vs control

Spontaneous

Mechanisms

oincreased PP1β phosphatase

ochronic dephosphorylation of cardiac connexin

Cardiac-restricted angiotensin-converting enzyme (ACE)

Overexpression with α-MHC Ang II concentration was 4.3-fold higher in ACE mice compared to WT

APD_90,_ phase 4 ↓

APD_50,_

phase 2 ↔

APD_75,_

phase 3 ↔

MAP90

Phase 4 ↓

MAP75

phase 3 ↓

MAP50_,_

phase 2 ↔

**A**PD_50,_ phase 2 ↓

APD_90,_ phase 4 ↓

β-AR mediated

APD_50,_

phase 2 ↑

APD_90,_ phase 4 ↓

Spontaneous

Other mechanisms

oIncreased I_Ks_ density

Spontaneous

Other mechanisms

oHypokalemia,

oReduced refractory period

Human cAMP-response element modulator (CREM) heart-directed

overexpression with α-MHC promoter

Spontaneous

Other mechanisms

oReduced phosphorylation of CREB and of PLB

oIncreased phosphorylation of SERCA2, PP1, and mRNA levels of ANP

Human cAMP-CREM heart-directed

Overexpression with α-MHC promoter

APD_25,_

phase 1 ↑

APD_50,_

phase 2 ↑

APD_90_

phase 4 ↑

Spontaneous and persistent

Other mechanisms

oLeaky SR Ca^2+^ stores

oDownregulation of connexin 40

Human cAMP- CREM and reduced RyR_2_-S2814A phosphorylation heart-directed

overexpression with germline transmission and Meox2-Cre crossing

Spontaneous at 3-month paroxysmal and persistent at 4–5 months

Other mechanisms

oincreased SR Ca^2+^ leak and CaMKII activity

oreduced connexin 40

Increased PR interval, AV block and

Wenckebach periodicity

Spontaneous

Other mechanisms

oreduced expression of connexin 40 and 43

oAng II signalling

Heart-restricted constitutively active Rac1 Rho

GTPase overexpression with α-MHC promoter

Spontaneous and persistent

Other mechanisms

oincreased NADPH oxidase activity

Spontaneous

Other mechanisms

oreduced protein expression of SERCA2a

oincrease expression of NCX protein

oβ_1_-adrenergic signalling

Heart-directed

overexpression of tumour necrosis factor-α with α-MHC promoter

APD_75_

Phase 4 ↔

Spontaneous

Other mechanisms

oimpaired Ca^2+^ loading

oreduced intracellular Ca^2+^ transients

Cardiac-specific overexpression of tumour necrotic

factor with α-MHC promoter

Spontaneous

Other mechanisms

oreduced connexion 40 expression

oinflammation

Spontaneous

Other mechanisms

oreduced SERCA2, increased ANP, BNP, βMHC, TGF-β1, TGF-β2, and TGF-β3

Reduced

nuclear envelope permeability by nucleoporin (NUP) 155 gene missense mutation on R391H

Spontaneous

Other mechanisms

oreduced HSP70 nuclear localization

SA and

AV nodes conduction defects

Spontaneous

Other mechanisms

olack of Ca_v_1.3, and reduced I_Ca,L_

Spontaneous

Other mechanisms

oreduced I_Ca,L_, Ca^2+^ transient amplitude, and SR Ca^2+^ content

↑ in atria

and ventricle

Prolonged PR intervals, double peak P-wave, and second and third degree

AV block

Spontaneous

Other mechanisms

oaltered expression of metabolic genes and K^+^ channels

oreduced HSP70

Melanin synthesis

enzyme dopachrome tautomerase global knockout

APD_50_, phase 2 ↔

APD_90_, phase 4 ↔

Spontaneous

Other mechanisms

oplasma membrane caveolae accumulation

oenlargement of mitochondria

APD_50_ phase 2 ↔

APD_80_ phase 4 ↔

Spontaneous

Other mechanisms

oincreased CaMKII-dependent phosphorylation of RyR2

oelevated SR Ca^2+^ leak

Overexpression of activated Gαq

cardiac protein with α-MHC promoter

APD_20_ phase 1, ↔

APD_50_ phase 2, ↔

APD_90_ phase 4, ↔

Spontaneous

Other mechanisms

oreduced expression of Pitx2,

oreduced expression of Nav1.5

oreduced expression of Kir2.1

Spontaneous

Other mechanisms

oreduced I_Ca,L_

oreduced Cav1.3 expression,

osignalling interaction between ankyrin-B and Cav1.2

Human sodium channel

Na_v_1.5 global missense mutation

APD_50_, phase 2 ↑

APD_90_, phase 4 ↑

Spontaneous

Other mechanisms

oreduced peak I_Na_

oincreased late I_Na_

↑ in atria

but not in ventricle

APD_50_, phase 2 ↔

APD_90_, phase 4 ↑

Spontaneous

Other mechanisms

oSR Ca^2+^ overload

oDADs

oincreased phosphorylation of RyR_2_

APD_30_, phase 2 ↔

APD_50_, phase 2 ↔

Spontaneous

Other mechanisms

oLack of FK506-binding protein 12.6

oDADs

oSR Ca^2+^ leak

oincreased I_NCX_

oCaMKII phosphorylation of RYR_2_ and PLB

Spontaneous

Other mechanisms

oincreased Ca^2+^ transient

AV block,

Bradycardia

Increased P-waves and QRS duration

Spontaneous

Other mechanisms

oreduced connexin 40 expression

Cardiac-specific AMPK-activating liver kinase B1

(LKB1) knockout with α-MHC promoter

✔ Intra-atrial

thrombi

Increased PR interval and

QRS duration in paroxysmal AF

Paroxysmal and persistent

Other mechanisms

oreduced expression of AMPK

oincreased in connexin 40 and 43 expression

oROS and inflammation

Human sodium channel

Na_v_1.5 cardiac-specific expression with α-MHC promoter

Premature ventricular

complexes and

non-sustained polymorphic VT

Spontaneous

Other mechanisms

oincreased late I_Na_

oincreased glycogen accumulation

omyofibril disorganisation

omitochondria injury

oNCX regulation of Na^+^ entry

Spontaneous

Other mechanisms

oAtrial cardiomyocyte produces calcitonin

oCalcitonin receptor and its ligand signalling governs fibroblast roles

oParacrine signalling between atrial cardiomyocyte released calcitonin and fibroblast

APD ↔

ERP ↔

Spontaneous

Other mechanisms

oPLK2/ERK/OPN is a dominant structural remodelling axis for AF generation

Mouse models that have been used to study the pathophysiology of AF, including atrial enlargement, electrophysiological alterations, apoptosis, functional and molecular underpinnings, and anatomical, transgenic; RYR2, ryanodine receptor 2; SR, sarcoplasmic reticulum; APD, action potential; SERCA mRNA, sarco/endoplasmic reticulum Ca^2+^-ATPase messenger ribonucleic acid; CTR, calcitonin receptor; KCNE1, potassium voltage-gated channel subfamily E member 1; AV, Atrioventricular block; MAP, monophasic action potential; PLB, phospholamban; ANP, atrial natriuretic peptide; β-AR, beta adrenergic receptor; PPβ1, protein phosphatase type 1β; NADPH, nicotinamide adenine dinucleotide phosphate; CaMKII, Ca^2+^/calmodulin-dependent protein kinase II; NCX, sodium–calcium exchanger; SERCA2a, Sarco/endoplasmic reticulum calcium (Ca^2+^) ATPase gene; TGF- β, Transforming growth factor beta; BNP, brain natriuretic peptide; HSP70, heat shock protein 70; DCM, dilated cardiomyopathy; AMPK, 5' adenosine monophosphate-activated protein kinase; PLK2, polo-like kinase 2; OPN, osteopontin; ERK1/2, extracellular signal-regulated kinase ½. ↔ unchanged in that condition; ✔ present in that condition; ↑ increased in that condition; ↓ reduced in that condition

## Introduction

AF is an irregular heart rhythm marked by chaotic atrial activation (fibrillatory waves on the ECG and loss of p wave) associated with irregular ventricular activation. It is the most common cardiac arrhythmia, a major clinical health problem, and a growing epidemic that manifests as a mixed disorder. It has been associated with familial inheritance due to a genetic mutation [1], can occur as ‘‘orphan’’ or idiopathic AF, and has been related to other cardiovascular diseases, underlying structural heart diseases such as cardiomyopathy [2] and most commonly to other risk factors, such as ageing [3, 4].

The incidence and prevalence of AF are rising globally [5]. The ageing population is a critical factor. The lifetime risks for development of AF were 1 in 4 at 40 years of age and above, and in the absence of antecedent congestive heart failure or myocardial infarction, the lifetime risks were 1 in 6 [6], indicating heart failure and myocardial infarction as myocardial substrate for the development of AF. Other non-modifiable risk factors in addition to ageing include sex, genetics, and race [7]. AF risk factors can also be classified as modifiable [8], and common modifiable risk factors of AF include physical activity, diabetes, obesity [9], obstructive sleep apnoea [10], alcohol [11, 12], and smoking.

Although the precise cellular and molecular mechanisms of AF remain unclear, they are purported to involve both structural and electrical remodelling of the atria, induced by the risk factors, to maintain vulnerable atrial substrate [7]. AF formation requires a vulnerable substrate and an initiating trigger. Atrial fibrosis promotes AF perpetuation by promoting localised re-entry through slowed atrial conduction. Circus movement, leading circle, spiral wave, and multiple wavelets have all been proposed as conceptual modes of re-entrant arrhythmia. The clinical relevance of these concepts is still uncertain as their real-world application has yielded highly variable results.

**Fig. 1:**
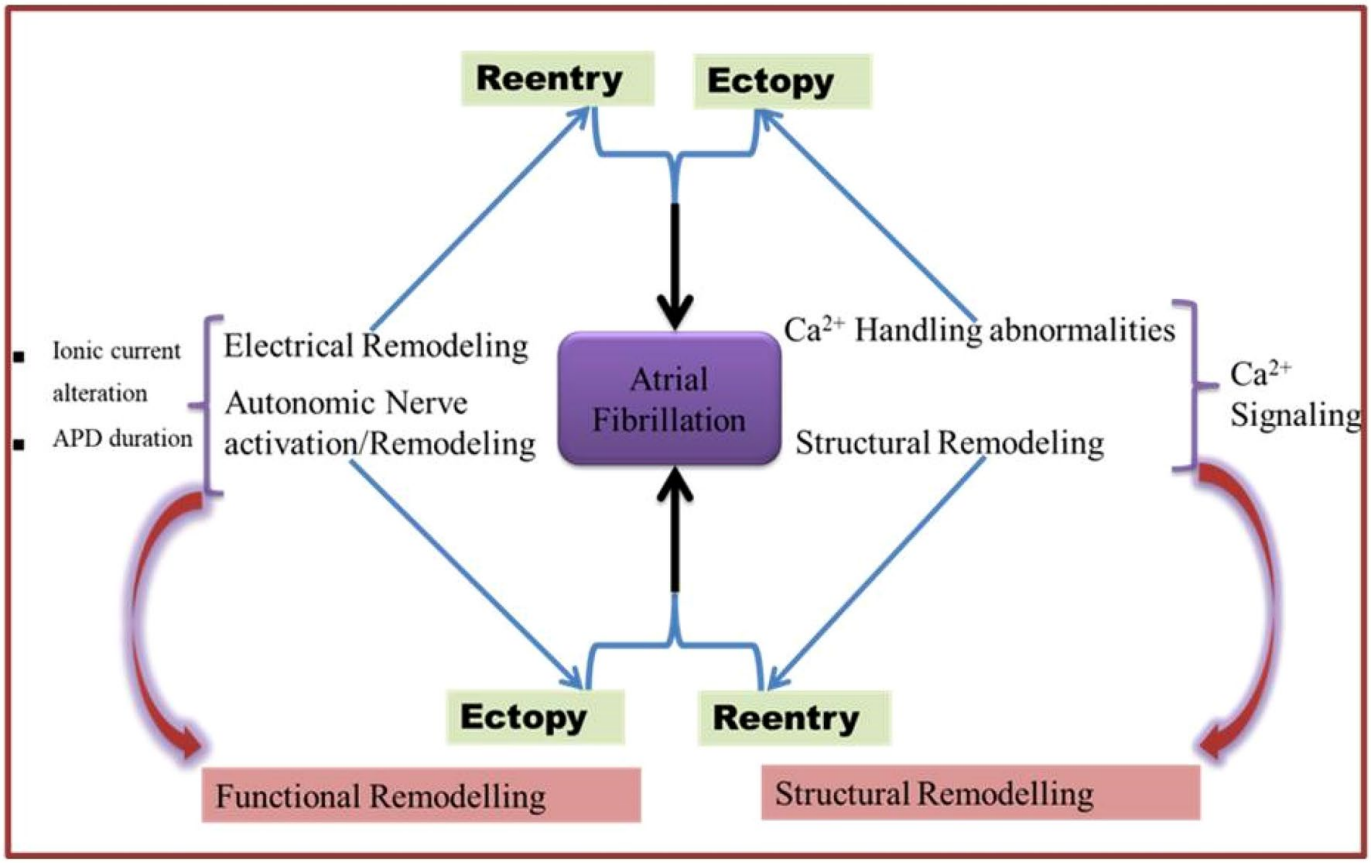
Conceptualised Mechanisms of Atrial Fibrillation showing functional and structural components of remodelling that maintain AF. Re-entry requires abbreviated action potential duration (APD) and/or conduction abnormalities. Ectopic firing occurs due to early after depolarization, delay after depolarization, and action potential prolongation. Changes in autonomic nerve activation produces significant and heterogeneous changes of atrial electrophysiology. Structural remodelling can be atrial enlargement and/or fibrosis atrial enlargement determine persistent AF through re-entry. Fibrosis distorts cellular architecture, extracellular matrix composition, and physical integrity of the atria. Ca^2+^ handling cause DADs

Changes in the physioanatomical properties of the atria are termed atrial remodelling. Pathological stimuli and perturbation of signalling like phosphatidylinositol 3-kinase and catalytic subunit alpha (PI3K[p110α]) cause both structural and electrical remodelling of the atria. This process involves changes in protein expression, collagen deposition, abnormal Ca^2+^ handling and contractility, and changes in ion current densities Fig. 1; [13]. Pharmacological attenuation of PI3K(p110α) activity caused late sodium current (late INa) stimulation to induce enhanced organelle sarcoplasmic reticulum Ca^2+^ load and QT interval prolongation [14]. Moreover, cardiac-specific inhibition of PI3Kα robustly eliminated angiotensin II time-dependent cell shortening and changes in L-type Ca^2+^ currents effects [15]. This effect was specific and large enough to approximately 90% in an order of magnitude. Pathophysiological evidence supports the role of PI3K(p110α) activity in AF susceptibility, AF-associated risk factors, and the cellular and molecular mechanisms that promote AF progression and perpetuation [13]. An endogenous reduction in the activity of PI3K(p110α) on a background of mammalian sterile 20-like kinase 1 (Mst1) resulted in a more severe cardiac phenotype. The model had enlarged atrial diameter, changes in the expression of potassium channels and metabolism-related genes, left atrial thrombi, extracellular matrix deposition, and spontaneous AF [16].

Atrial enlargement is a clinical predictor of AF [17]. In about forty (40) transgenic and knockout mouse models of AF currently existing, of which some were created based upon knowledge gained from clinical mutation analysis of arrhythmias, including PI3K(p110α) transgenic mouse model, over 70% of them reporting atrial size showed enlarged, greater atrial size, or mass (Table 1). Several observational studies have identified increase in atrial size and cardiac stretch a mechanism of AF in humans [18–20]. Nonetheless, information is clearly required from further studies to elucidate the determinants of atrial enlargement, which are poorly understood – with a potential to increase our knowledge of pathophysiology of AF, and identify novel therapeutic targets. Starting with the types of AF and a model of remodelled atrial tissue, this review provides an overview of the potential roles of PI3K(p110α) gene, a molecular regulator of cell and organ size, in the induction of cardiac-specific pathological atrial enlargement and in physiological mechanisms of AF progression and maintenance.

## Types of AF and a model of remodelled atrial tissue

AF has been studied for over a century and the mechanism is evolving. AF can be classified clinically into different types based on duration, frequency of episodes, and manifestation. This includes i) single episode or ‘lone’ AF, ii) paroxysmal, iii) persistent, iv) long-standing persistent, and v) permanent [21].

(i) First episode- original episode of AF previously undiagnosed regardless of presence and/or severity of AF-related symptoms.

(ii) Paroxysmal- AF that terminates spontaneously, usually within 48 h. However, some episodes may persist for up to 7 days.

(iii) Persistent AF- episodes that last beyond 7 days. This type of AF is generally not self-terminating.

(iv) Long-standing persistent- episodes that last for greater than one year.

(v) Permanent AF- describes AF that is not self-terminating and does not respond to treatment or medication.

It should be noted that these definitions to some extent represent an artificial characterisation of AF syndromes for the purposes of clinical categorization particularly in the context of clinical studies. In reality, there is a spectrum of AF phenotype severity ranging from ‘lone’ AF to permanent AF.

Symptoms of AF include palpitations, fatigue, psychosocial distress, breathing difficulties, chest tightness, sleeping difficulties, and poor quality of life [22–24].

The underlying mechanisms of AF are still incompletely understood. An important feature of AF is very rapid and chaotic atrial activation, which can be caused by re-entry activity or spontaneous foci ectopy. AF requires re-entry and focal ectopic trigger, predominately arising from the pulmonary veins [25]; however, non-pulmonary vein triggers are also well described [26]. The initiating triggers and re-entry in addition to vulnerable atrial substrate such as atrial enlargement perpetuate AF.

## Physiological mechanisms of AF and PI3K(p110α)/class IA PI3K

In the remodelled model of atrial tissue and as a physiological process, ectopic triggers (repetitive depolarization) can be due to early after depolarisations (EADs) or delay after depolarisations (DADs). EADs occur at the plateau phase (phase 2) or phase 3 of action potential duration, whereas DADs occur at phase 4 of action potential repolarisation. Triggers are regarded as abnormal secondary repolarisations and occur when DADs or EADs reach the threshold potential. Whereas, EADs are believed to be caused by slowing of repolarization, DADs are known to be caused by abnormal diastolic Ca^2+^ release by ryanodine receptor 2 (RyR2).

Trigger is associated with the development of arrhythmias through alterations in action potentials (APs). Enhanced sympathetic tone increase the probability of EADs. To assess the possibility of PI3Ks-induced trigger, APs were measured at different pacing frequencies in presence of increased sympathetic tone with isoproterenol (ISO), under PI3K inhibition. Control canine myocytes exposed to ISO but not class IA PI3K inhibitors had no EADs, but a decrease in APD and AP plateau height compared with untreated cells. In contrast, in the presence of 50 nM or 500 nM PI-103, ISO induced EADs in the ventricular myocytes [27]. In contrast to Lu et al., we did not observe EADs either in wild-type or Akita right atrial myocytes with reduced PIP3 signalling, in the presence of 1 µM ISO [28]. Together, the atria have both parasympathetic and sympathetic innervations, unlike the ventricle, which could be offsetting the effect of each other. It could also be that sympathetic response of the atria may differ from that of the ventricle, and in the presence of enhance sympathetic response, direct inhibition of class IA PI3K (comprising a catalytic subunit PI3K(p110α), PI3K(p110β), or PI3K(p110δ) and a p85 regulatory subunit might predispose to arrhythmias. Indeed, experimental studies in preclinical models show the essential roles of PI3Kα in the regulation of Na^+^ channel activity, control of the arrhythmias, and cardiac safety [29]. Although, the specific role of PI3K(p110α)-induced trigger event remains to be investigated in better details, in reduced PI3K signalling and diabetes there was slow repolarisation in both the atria and ventricle [27, 28]. The electrophysiological feature, in part, anchors rotors and wave breaks fibrillatory activities in the presence of EADs in a mouse model with spontaneous and sustained AF and enhanced persistent Na + current due to a mutation in NaV1.5 channel [30].

Besides trigger, arrhythmia at tissue level is propagated by re-entry. The concepts of re-entrant mechanisms of AF have been proposed by the elegant of works of Garrey [31, 32], Moe [33], and amongst others [34, 35], to include circus movement, leading circle, spiral wave, and multiple wavelet. Detailed discussion of these concepts is beyond the scope of this work, but these examples support their role in arrhythmogenesis – particularly, wavelength shortening and reduced conduction velocity or refractory period are present in the enlarged and remodelled atria leading to sustained re-entrant-based tachycardias [36]. Atrial enlargement as a clinical correlate of AF helps to promote AF by favouring more wavelet formation [37]. Essentially, constitutive activation of PI3K(p110α) protein-induced cardiac hypertrophy [38] and cardiac hypertrophy induces atrial and ventricular arrhythmias [39], through alteration in cardiac ion channels. In particular, dominant negative PI3K(p110α) expression has been associated with greater atrial size [16].

## PI3K(p110α) mediates atrial size and AF

*Drosophila* having PI3K(p110α) deficiency have small cells and organs [40]. Likewise, mice deficient for cardiac-specific PI3K(p110α) expression displayed small hearts, whereas those with enhanced cardiac-specific PI3K(p110α) expression displayed large hearts [41]. These data demonstrate the importance of the PI3K regulatory pathway in physiological cell and organ growth response in invertebrate and vertebrate animals.

Atrial hypertrophy is an important feature of adverse atrial remodelling as atria respond to pathological stimuli, such as myocardial stretch. Atrial dilation, as well as enlargement, is associated with AF ([42]; Table 1) and left atrial size is a known risk factor for the development atrial fibrillation [43]. Although, there is significant lack of literature on atrial hypertrophy and chamber-specific mechanisms of hypertrophy are largely unknown, atrial and ventricular hypertrophy may have comparable mechanisms. PI3K(p110α) is a key molecular regulator of cardiac size [41], through exercise (physiological) and aortic banding (pathological) [38]. Physiological and pathological hypertrophy due to PI3K(p110α) transgene are distinct based on molecular underpinnings. Whereas physiological hypertrophy is associated with normal function, pathological hypertrophic is associated with adverse effects. Pathological atrial hypertrophic remodelling is a multiplex process involving myofibroblast differentiation, cardiac myocyte growth, and loss of myofibril content [44].

Atrial enlargement [45] as well as fibrosis [46] are important players in AF progression. Left atrial diameter and volume stratification are an assessment for follow-up surveillance to detect AF in the clinics. Furthermore, mapping and removal of fibrotic areas and homogenisation of scares are currently emerging as rhythm control measures for AF patients. Pretorius et al. demonstrated atrial fibrosis and enlargement and increased susceptibility to AF in mice with reduced PI3K activity in the heart and Mst1 [16]. Combined assessment of left atrial fibrosis and size facilitates the identification of patients with better ablation success potential [47].

**Fig. 2:**
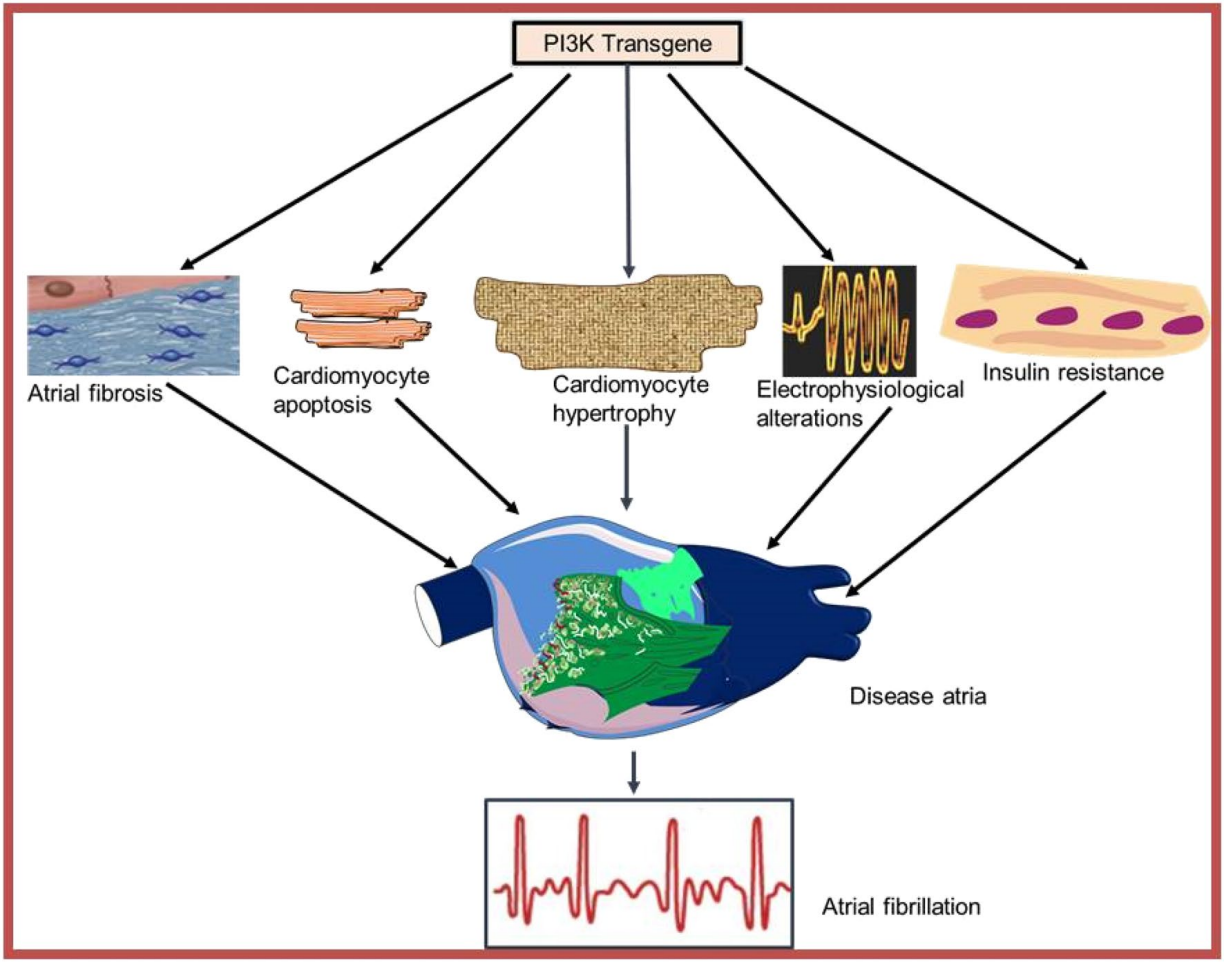
A schematic of atrial hypertrophy, fibrosis, apoptosis, electrophysiological alterations, and insulin resistance as PI3K(p110α) transgene hypofunction phenotypes leading to irregular heartbeat, disease atria, and atrial fibrillation

Although, atrial enlargement is an important mechanism of AF [48], the details of the molecular mechanisms of atrial size control and AF susceptibility are unknown. Atrial enlargement is part of the cellular remodelling that produces atrial substrate and AF and indicates elevated pressure and/or higher than normal blood volume in the atria. Bruton’s tyrosine kinase, a Tec family tyrosine kinase, an effector of PI3K activity, whose activation, in part, depends on the binding of PtdIns(3,4,5)P3 to the PH domain and is important for an enhanced intracellular Ca^2+^ signalling, caused AF in an off-target side effect, through atrial enlargement. A daily dose of selleckchem (ibrutinib), a non-specific Bruton tyrosine kinase inhibitor intraperitoneally injected for 4 weeks in mice, produced spontaneous AF, left atrial enlargement, myocardial fibrosis, and increased inflammation accompanied by prolonged atrial effective refractory periods without profound alteration in the action potential duration [49]. Although the effects were present in mice without Bruton tyrosine kinase, mice that received acalabrutinib, a specific Bruton tyrosine kinase inhibitor for 4 weeks had AF, showing an off-target side effect [49]. Chemoproteomic profiling of ibrutinib in cardiac tissue, where homogenised cardiac tissues were incubated with a biotinylated acylphosphate ATP derivative to transfer biotin to the conserved lysine residues in the ATP-binding pocket of protein kinases and other ATP-binding proteins for longer periods, identified BTK, proto-oncogene tyrosine-protein kinase (FYN), mitogen-activated protein kinase kinase 5 (MEK5), C-terminal Src kinase (CSK), and receptor-interacting serine/threonine kinase 3 as the potential targets of ibrutinib [49]. When the experiment was repeated with acalabrutinib, a second-generation ibrutinib, BTK and RIPK3 were rather identified as the targets [49]. These assessments when comparatively analysed by the authors limited the potential candidates of ibrutinib-associated AF-inducible targets to FYN, MEK5, and CSK. Consequent genetic manipulation of the three kinases in mice led to the final identification of Csk inhibition, as the mechanism of ibrutinib-associated AF, as cardiac-specific Csk knockout in mice, mimicking ibrutinib treatment predisposed to increased AF, left atrial enlargement, fibrosis, and inflammation [49]. PI3K(p110α) deficiency in mouse heterozygous for PI3K(p110α) transgene might reduce stress-induced dilation in dilated cardiomyopathy. Surprisingly, the double transgenic mouse model heterozygous for PI3K(p110α) on a background of Mst1 overexpression had AF and adverse atrial enlargement as assessed by echocardiography [16]. This is in contrast to the overexpression of PI3K(p110α) and Mst1 [16], suggesting a role for PI3K(p110α) heterozygous in atrial enlargement. To gain better insight into PI3K(p110α)-induced atrial greater size, a complete knowledge of the PI3K(p110α)-dosing effect in form of the heterozygous and homozygous transgene is required. This will improve the understanding of the likely critical roles of PI3K transgene in the control of atrial size, muscle mass, and atrial disease (Fig. 2).

Consistent to the upper chamber of the heart, PI3Kα-dominant negative mutant mice with heart failure pressure overload had dilated cardiomyopathy, by increased gelsolin-mediated actin severing activities in vivo. Adult cardiac stretch in PI3Kα deficiency perturbed sarcomeric actin cytoskeleton. The actin remodelling from the biomechanical stress stimuli mechanotransduction was prevented by PIP3, produced upon PI3Kα activation in feedback response. The gelsolin-driven actin cytoskeletal remodelling (depolymerisation) in heart failure was mechanistically underlined by increased expression of atrial and beta natriuretic peptides and increased cross-sectional areas of cardiomyocytes and chamber dilation [50]. The profound pathology was attenuated at the PI3Kα mutant background deletion of gelsolin [50], a Ca^2+^-dependent protein that regulates the dynamics of actin filament assembly and organisation and extensively expressed in many tissues, including heart, brain, and immune cells. Hence, PI3Kα/PIP3 are negative regulators of gelsolin activity. Furthermore, in experimental myocardial infarction, PI3Kα activity necessitated endothelial cell and cardiomyocytes hypertrophic response [51]. In this setting, pharmacological ablation of PI3Kα led to worsened cardiac dysfunction, profound apoptosis and inflammation, and suppressed Akt/glycogen synthase kinase 3β/endothelial nitric oxide synthetase signalling, as well as hypertrophy, post-MI [51]. In cell-specific manner, genetic PI3Kα inhibition in endothelial cells reduced coronary blood vessel density and in cardiomyocytes resulted in moderate cardiac systolic dysfunction at baseline [51]. Although these findings are novel and counterintuitive to the concept of PI3Kα hypofunction in atrial mass and enlargement and reveal potential PI3Kα inhibition cardiotoxicity, notably, cardiac hypertrophic risk of PI3Kα is dose dependent of its activity, relies on cell-specific communication effects and paracrine signalling, and has not been completely deciphered in better details.

## Molecular mechanism of PI3K(P110α)-induced atrial enlargement

Several elegant studies show that cardiac cells require active PI3K/Akt signalling to maintain proliferation. Mice homozygous for 110-kDa catalytic subunit isoform (Pik3cα), demonstrating loss of expression of PI3K(p110α), had embryonic lethality at day 9.5 due to a severe defect in the proliferative capacity of the embryo. The defect was demonstrated by the observation that the mouse embryonic fibroblasts from the explants of PI3K(p110α) homozygous embryos but not those of wild-type and the PI3K(p110α) heterozygous embryos failed to replicate in Dulbecco’s modified Eagle’s medium and foetal calf serum, even with supplemental growth factors [52]. How dose-dependent (heterozygous and homozygous) effects of PI3K(p110α) may regulate atrial cells size leading to AF is unclear. We know that PI3K biological signalling network maintain cell viability and proliferation, reduce apoptosis, and respond to constantly changing external and internal conditions to maintain dynamic equilibrium state. When the signalling is adjusted by way of dosing, the network could be acutely or chronically altered. For instance, chronic stimulation of tissue-resident cells with growth factors can cause aberrant shift from resting to actively proliferating cells.

**Fig. 3:**
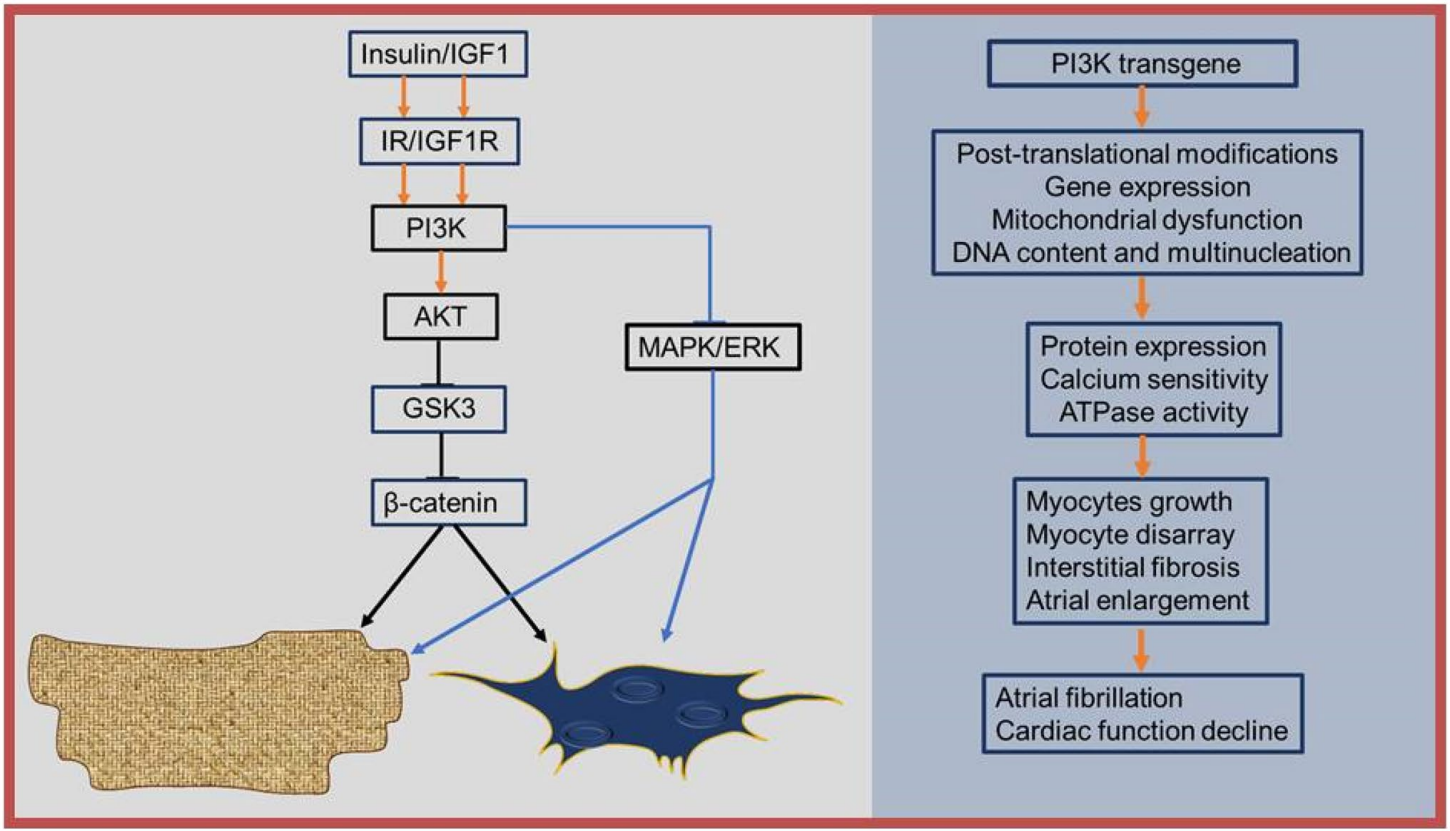
Mechanisms of PI3K(p110α)-induced atrial enlargement. Whereas, inactivation of PI3K would result in the nuclear accumulation of GSK3, and GSK3 inhibition by PI3K activation on the other hand or p38 can mediate β-catenin activity to regulate cell cycle activity, DNA content, and nucleation. Cell cycle activity, DNA content and nucleation, and mitochondria dysfunction that result from an initial molecular activity of a PI3K transgene hypofunction would be guided by post-translational modification and gene expression to myocyte growth, disarray, and fibrosis—phenotypical features and AF (right). PI3K(p110α)-induced atrial enlargement can also occur through Ang II-induced TGF-β1 release in enlargement cardiomyocytes to trigger paracrine signalling between the cardiomyocytes and fibroblast leading to proliferation of fibroblast and further enlargement, structural and morphological alteration before AF (left)

In response to growth factor receptor activation, PI3K(p110α) signalling begins leading to the synthesis of phosphatidylinositol (3,4,5)-trisphosphate (PIP3) from phosphatidylinositol (4,5)-trisphosphate (PIP2) and translocation of Akt to cell membrane. Phosphorylation and activation of Akt leads to inactivation of tuberous sclerosis (TSC) 1 and 2 and activation of Ras homolog enriched in brain (Rheb) and the mammalian target of rapamycin (mTOR1). Through this process, PI3K and its downstream signalling effectors, such as Akt, PIP3, mammalian target of rapamycin, GSK3, and PDK1, regulate cell growth and survival [53]. It has been suggested that PI3K promotes cardiac cell proliferation through the inhibition of the GSK3 and mitogen-activated protein kinases/extracellular signal-regulated kinase (MAPK/ERK). This is consistent with the finding that PI3K signalling is accentuated during suppression of MAPK activation in stress-related growth of neonatal heart [54]. PI3K activation leads to inhibition of GSK3. Downstream GSK3 inhibition as a consequence leads to activation of D- and E-type cyclins, glycogen synthase, mTORC1, and nuclear factor of activated T-cells, a regulator of hypertrophy. GSK3 inactivation additionally occurs through p38. GSK3 regulates the canonical Wnt signalling. β-catenin activation by GSK3 results in ubiquitination and degradation of β-catenin by proteasome to stop gene expression. β-catenin is stable and translocates to the nucleus, when GSK3 is inhibited, resulting in gene expression. β-catenin modulates a host of events through fibroblast differentiation and fibrosis to cardiomyocyte hypertrophy (Fig. 3, right).

Furthermore, PI3K(p110α) transgene-induced hypertrophy is a feature of stretched cardiomyocytes and stretch as pathological stimuli results in angiotensin II (Ang II) release, which triggers the activation of transforming growth factor beta (TGF-β) [55], leading to a series of transcriptomic changes and post-translational modification and phenotype manifestation (Fig. 3, left). Cells interact with one another within their environment of occupation, through paracrine signalling that favours fibrosis. How the cells respond to the signalling may differ depending on cell type. For instance, Ang II-induced TGF-β release in cardiomyocyte may lead to cardiomyocyte death and hypertrophy, whereas in fibroblast, it may cause fibroblast proliferation. Cardiomyocyte death and hypertrophy and fibroblast proliferation lead to fibrosis and regeneration [56].

## PI3K(p110α) as a target for prevention of atrial enlargement

Conventional therapy for cardiac arrhythmias is limited and it is time to think of biological therapies (gene therapy, cell therapy, or both), as an alternative to the present therapeutic regime, which rely on pharmacology or resource heavy interventional approaches [57]. Gene therapy is the use of genetic material to modify the genetic codes of the cell of the patient carrying inherited and/or acquired disease by transfer of genetic material into that cell for cure or to improve function. The genetic material can be transferred through nanoparticles, vectors, or plasmids to target specific traits of a disease. This approach may present advantage to AF management because the method can be tissue specific with minimal or no off-target effects. However, AF is a mixed disorder and single gene modification may not be insufficient even in the setting of a valid therapeutic target. Nonetheless, a single gene validated to have pleotropic effect on the numerous substrates for AF may alleviate the challenge and be a good choice for gene therapy for AF. The cardio protective role of PI3K(p110α) could be utilised to customise therapy for AF, particularly in this era of personalised medicine. Based on our molecular understanding of the atrial substrates and AF pathophysiology, gene therapy targets for AF include atrial enlargement, apoptosis, fibrosis, hyper innervation of the autonomic nervous system, ion channel, and gap junction alteration. As discussed above, constitutive PI3K(p110α) expression attenuates the targets to ensure cardio protection, highlighting a potential non-pharmacological relevance of a moderate dose of PI3K(p110α) gene in the pathological atrial remodelling. It is therefore mechanistically feasible that PI3K(p110α) gene may prevent atrial enlargement when identified as a risk factor, even before conventional treatment is required. The attractive potentials, nonetheless, drawbacks can be foreseen for a cardiac-targeted PI3K(p110α) gene therapy. PI3K-targeted gene therapy might be complicated with respect to impacts on genes of the targeted cells, delivery and activation, and immune system response. Although, drugs can be given to temporarily suppress the immune system response and lowest doses of effective viruses or viruses with reduced susceptibility to cause immune response can be used, it is still a concern—with a potential to cause debilitating illness or even death that immune systems fight to ward off foreign matters, such as bacteria and viruses, when introduced in the system. Introduced gene moulds itself to become a permanent part of an entire genome. This process can disrupt another gene or lead to an inappropriate location of the gene. Unguided delivery, activation, and integration of the PI3K gene to unspecific places of the genome can occur and would be carcinogenic. The role of PI3K in carcinogenesis is well known.

## Conclusion

Pathological increase in atrial muscle size, otherwise known as ‘‘atrial enlargement’’ is a mechanism of AF. AF consequently induces atrial enlargement, suggesting a process through which AF promotes itself. Individuals with minimal to severely dilated atria may be more likely to develop AF than those with normal atrial size. A reduction in atrial size with gene therapy as a non-interventional therapy will be associated with a reduced AF burden. It will also be associated with primordial prevention of AF, suggesting huge potential in identify and treat risk factors (i.e. risk factor prevention), before the disease occurs. A better understanding of AF molecular mechanisms is required to improve treatment strategies and management of AF. Evidence for molecular mechanisms of PI3K(p110α)-induced atrial enlargement as a clinical correlate of AF is crucial, and studies elucidating cellular mechanisms of atrial enlargement are needed. Advancing our knowledge of the role of PI3K(p110α) gene in the symptoms, pathophysiology, AF-associated risk factors, and in the incidence of AF will help to provide new preventive and treatment measures and reduce the public health burden of AF. The works reviewed in this study highlight that PI3K(p110α) is very likely a master regulator of atrial size, yet its implications remain to be defined with respect to atrial size control and therapeutic strategies for AF management.
